# Chromosomal instability and inflammation: a catch-22 for cancer cells

**DOI:** 10.1007/s10577-023-09730-y

**Published:** 2023-08-10

**Authors:** Anouk van den Brink, Maria F. Suárez Peredo Rodríguez, Floris Foijer

**Affiliations:** European Research Institute for the Biology of Ageing, University of Groningen, University Medical Center Groningen, Antonius Deusinglaan 1, 9713 AV Groningen, The Netherlands

**Keywords:** Chromosomal instability, Aneuploidy, CIN-induced inflammation

## Abstract

Chromosomal instability (CIN), an increased rate of chromosomal segregation abnormalities, drives intratumor heterogeneity and affects most human cancers. In addition to chromosome copy number alterations, CIN results in chromosome(s) (fragments) being mislocalized into the cytoplasm in the form of micronuclei. Micronuclei can be detected by cGAS, a double-strand nucleic acid sensor, which will lead to the production of the second messenger 2′3′-cGAMP, activation of an inflammatory response, and downstream immune cell activation. However, the molecular network underlying the CIN-induced inflammatory response is still poorly understood. Furthermore, there is emerging evidence that cancers that display CIN circumvent this CIN-induced inflammatory response, and thus immune surveillance. The STAT1, STAT3, and NF-κB signaling cascades appear to play an important role in the CIN-induced inflammatory response. In this review, we discuss how these pathways are involved in signaling CIN in cells and how they are intertwined. A better understanding of how CIN is being signaled in cells and how cancer cells circumvent this is of the utmost importance for better and more selective cancer treatment.

It is well known that tumors are heterogeneous in nature, both between tumors (intertumoral heterogeneity) or within a single tumor (intratumoral heterogeneity). One of the processes fueling intratumor heterogeneity is chromosomal instability (CIN) (Bakhoum and Landau 2017). CIN is defined as an increased frequency of chromosomal missegregation over successive cell divisions. A direct consequence of CIN is abnormalities in chromosome structure and/or altered chromosome (arm) copy numbers. The latter is also referred to as aneuploidy (Sheltzer and Amon 2011; Santaguida and Amon 2015; Schukken and Foijer 2018; Chunduri and Storchová 2019). Aneuploidy and CIN are interrelated, but not the same. CIN is the *process* that leads to increased missegregation events that yields cells with an aneuploid *state*. Distinguishing between CIN and aneuploidy is key to understand their independent contributions to tumor evolution and growth. CIN typically yields a heterogenous tumor cell population and provides cells with an ability to undergo selective evolution. However, when CIN rates are low, tumors can still be highly aneuploid with very little heterogeneity, potentially reducing their evolutionary capacity (Bakhoum and Compton 2012). CIN and aneuploidy are well tolerated in most human tumors, as reflected by their occurrence in the majority of human cancers (Sheltzer and Amon 2011; Carter et al. 2012) and their association with poor patient prognosis (Carter et al. 2006; Walther et al. 2008; Orsetti et al. 2014), metastasis (Bakhoum et al. 2018; Li et al. 2021), tumor aggressiveness (Carter et al. 2006; Orsetti et al. 2014), and therapy resistance (Lee et al. 2011; Ippolito et al. 2021). However, CIN but also stable aneuploidy have detrimental effects on the survival of untransformed cells. The proliferation defects of untransformed cells with CIN or (stable) aneuploidy result from the multiple converging stress signaling pathways induced by either of them, including proteotoxicity, metabolic stress, and inflammatory response. This paradox between the response of tumor cells and untransformed cells to aneuploidy is referred to as the aneuploidy paradox and suggests that tumor cells have developed mechanisms to cope with these stresses induced by CIN and the resulting aneuploidy (Sheltzer and Amon 2011; Siegel and Amon 2012; Zhu et al. 2018; Zhou et al. 2020).

Work from the past few years has revealed an important role for cancer cell-intrinsic inflammatory signaling resulting from CIN. The innate immune signaling pathways cGAS/STING and RLR/MAVS, both originally described as innate defense mechanisms against pathogens (Ablasser and Hur 2019), were found to play a prominent role in eliciting this tumor cell-intrinsic inflammatory response (Hong et al. 2019; Beernaert and Parkes 2023). This tumor cell-intrinsic inflammatory signaling was reported to increase the immunogenicity of tumors with CIN, thereby enhancing immune cell recruitment, early tumor detection, and tumor cell clearance (Santaguida et al. 2017; Wang et al. 2021). However, other studies point toward a more complicated relationship between CIN and immune surveillance, as CIN has been associated with immune evasion rather than immune surveillance in the context of tumorigenesis (Davoli et al. 2017; Schubert et al. 2021; Li et al. 2021). This suggests that tumors with CIN have evolved more complex mechanisms to adjust or use CIN-induced inflammation in a pro-tumorigenic manner. Here, we review the intertwined relationship between CIN and inflammation in cancer by describing the intratumoral mechanisms involved in CIN-induced inflammation and their consequences for the survival of tumors with CIN.

## Triggers for CIN-induced inflammation

**Fig. 1:**
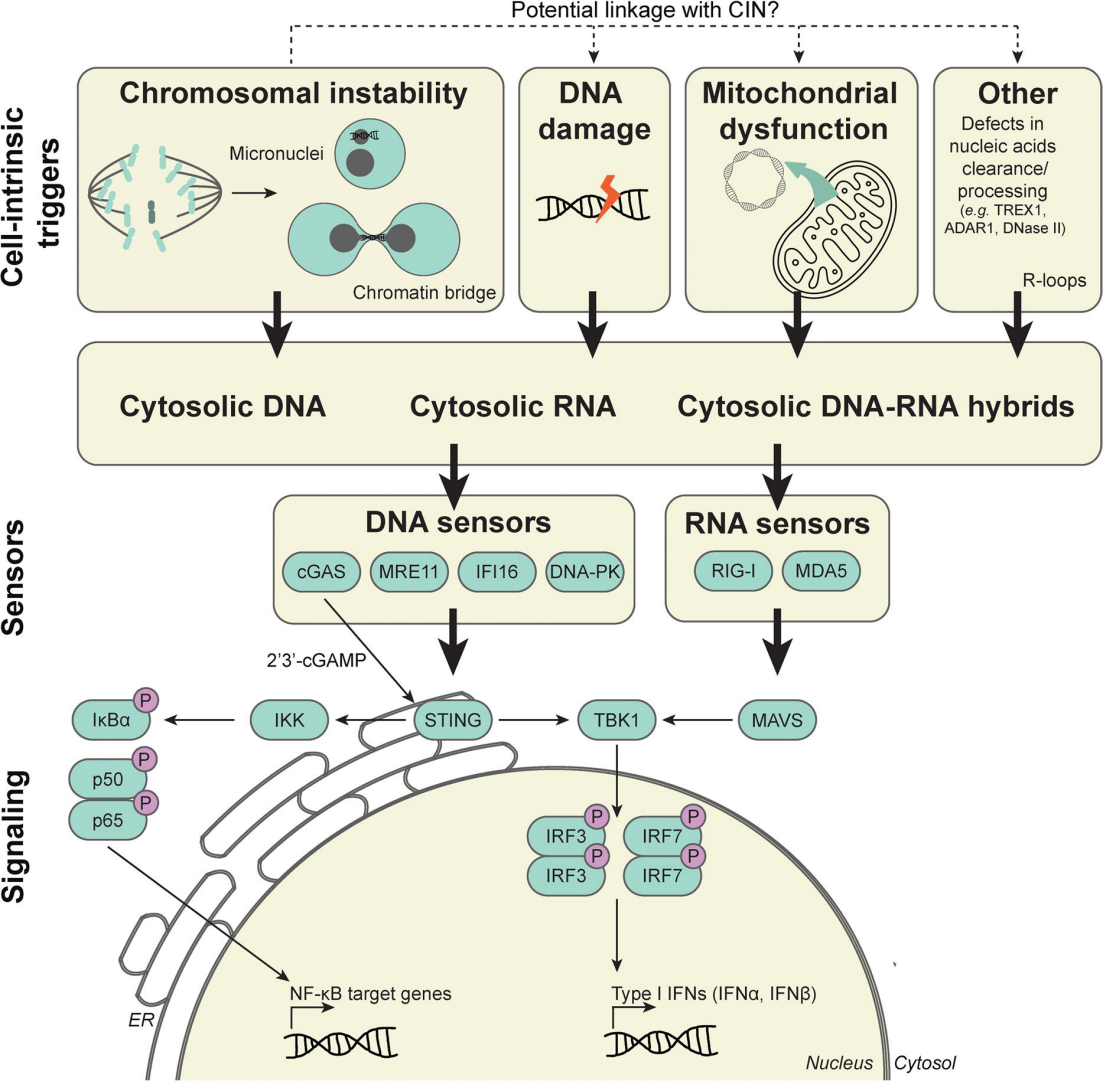
An overview of the various triggers for tumor cell-intrinsic inflammatory signaling and the downstream response. Various cell-intrinsic triggers can result in the release of endogenous DNA, RNA, or DNA-RNA hybrids in the cytosol, including chromosomal instability, DNA damage, mitochondrial dysfunction, defects in nucleic acid clearance/processing, and R-loops. These “out-of-place” nucleic acids activate DNA and/or RNA sensors, resulting in inflammatory signaling. Consequently, the expression of type I IFNs and NF-κB target genes are induced. *ADAR1, adenosine deaminase acting on RNA 1; cGAS, cyclic GMP-AMP synthase; 2*′*3*′*-cGAMP, cyclic GMP-AMP; DNA-PK, DNA-dependent protein kinase; IFI16, interferon-γ inducible 16; IFN, interferon; IKK, IκB kinase; IRF3/7, interferon regulatory factor 3/7; MAVS, mitochondrial antiviral signaling protein; MDA5, melanoma differentiation-associated protein 5; MRE11, meiotic recombination 11; NF-κB, nuclear factor κ-light-chain-enhancer of activated B cells; RIG-I, retinoic acid-inducible gene-I; STING, stimulator of interferon genes; TBK1, TANK-binding kinase 1; TREX1, three prime repair exonuclease 1*

One of the mechanisms underlying the CIN-induced inflammatory response involves activation of the innate immune DNA sensing pathway cyclic GMP-AMP synthase (cGAS)/stimulator of interferon genes (STING) (Fig. 1), extensively reviewed before (Hong et al. 2019; Beernaert and Parkes 2023). The trigger for activation of the cGAS/STING signaling pathway in the context of CIN is genomic double-stranded DNA (dsDNA) in the cytosol, for instance, from micronuclei that arise when chromosomes lag during anaphase. Rupture of the micronuclear membrane results in exposure of genomic dsDNA to the cytosol, which acts as a ligand for the innate immune dsDNA sensor cGAS. cGAS activation leads to the production of the second messenger 2′3′-cGAMP, which in turn activates STING and IRF3-dependent expression of type I interferon genes downstream (MacKenzie et al. 2017).

Although dsDNA in micronuclei is a well-established trigger that activates cGAS/STING signaling, recent studies suggest that other mechanisms might also trigger inflammation as a result of CIN. For example, Flynn *et al*. demonstrated that cGAS and a downstream type I interferon response are activated by chromatin bridges more than by micronuclei (Flynn et al. 2021). Furthermore, mitotic arrest, for instance, induced by taxanes, can also enhance cGAS activity and depends on association of cGAS with mitotic chromosomes. However, the downstream accumulation of phosphorylated IRF3 in a cGAS-TBK1-dependent manner promotes cell death rather than an inflammatory transcriptional response in the latter case (Zierhut et al. 2019).

It is becoming increasingly clear that the characteristics of genomic dsDNA (in the cytosol) affect its ability to mount a cGAS or other DNA sensor-dependent cell-intrinsic inflammatory response. For instance, Zierhut *et al.* demonstrated that nucleosome-bound DNA is less effective in activating cGAS activity than naked DNA (Zierhut et al. 2019). Furthermore, recent work suggests that CIN is associated with changes in both chromatin accessibility and transcription resulting from micronuclei formation (Agustinus et al. 2023; Papathanasiou et al. 2023). In agreement with this, epigenetic modifications, such as H3K79 methylation, histone H3 acetylation, and chromatin organization in micronuclei were found to determine recognition by cGAS and its downstream response (MacDonald et al. 2023; Agustinus et al. 2023). Finally, as CIN can drive many different types of mitotic abnormalities that each could well impact the structure of genomic DNA differently (Lee et al. 2013), each mitotic abnormality might influence the inflammatory response in a different manner (Flynn et al. 2021). This might apply for the methods used to induce CIN in the lab, but importantly, for the drivers of CIN in primary cancers as well.

### Potential other sources of CIN-induced inflammation

In addition to cytosolic DNA originating from the nucleus, other triggers can also activate tumor cell-intrinsic inflammatory signaling, such as mitochondrial DNA (West et al. 2015; Luzwick et al. 2021; He et al. 2022), DNA-RNA hybrids derived from R-loops (Crossley et al. 2022), and endogenous (mitochondrial) dsRNA (Tigano et al. 2021; Zhang et al. 2022; de Reuver et al. 2022; Hubbard et al. 2022; Jiao et al. 2022) (Fig. 1). Detection of these structures does not only rely on the cGAS/STING signaling pathway, but also on other DNA sensing and RNA-sensing RLR/MAVS signaling pathways. Some of these structures might have a direct link to CIN. For example, R-loops have been reported to enhance replication stress, which is associated with increased rates of mitotic abnormalities (Gan et al. 2011; Panatta et al. 2022). Although R-loops on their own trigger cell-intrinsic inflammatory signaling (Crossley et al. 2022), the elicited inflammatory response might differ from the response induced by CIN. Nevertheless, some of these structures could well contribute to the cumulative CIN-induced cell-intrinsic inflammatory response, as CIN elicits pleiotropic stresses, including proteotoxicity and metabolic stress (Zhu et al. 2018; Zhou et al. 2020).

## The downstream consequences of CIN-induced cell-intrinsic inflammatory signaling

**Fig. 2:**
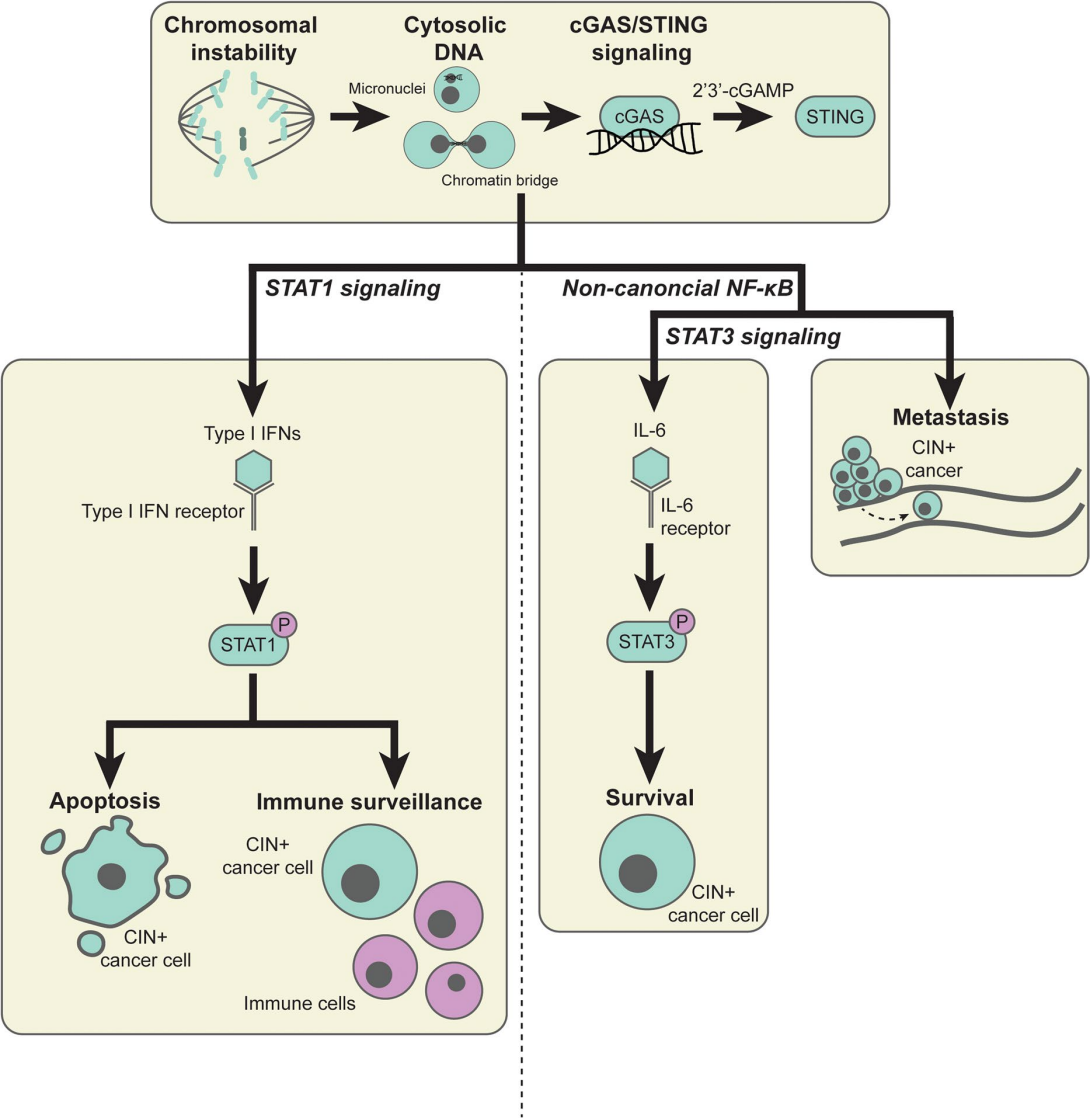
The intertwined STAT1, (non-canonical) NF-κB, and STAT3 response downstream of CIN-induced cGAS/STING signaling. CIN-induced cGAS/STING signaling has been reported to activate the STAT1 and STAT3 network as well as canonical (not shown here) and non-canonical NF-κB signaling. CIN-induced STAT1 signaling has a tumor suppressive role, as it promotes cancer cell apoptosis and immune surveillance. On the contrary, CIN-induced non-canonical NF-κB and STAT3 signaling are considered pro-tumorigenic, as these signaling pathways promote CIN^+^ cancer cell survival and metastasis. *2′3′-cGAMP, cyclic GMP-AMP*; *cGAS, cyclic GMP-AMP synthase; IFN, interferon; IL-6, interleukin-6; NF-κB, nuclear factor κ-light-chain-enhancer of activated B cells; STAT, signal transducer and activator of transcription; STING, stimulator of interferon genes*

CIN-induced cell-intrinsic inflammatory signaling appears to have tumor-suppressive as well as tumor-promoting effects that are likely context dependent and might even co-occur in single tumors. The Janus kinases (JAK)/signal transducer and activator of transcription (STAT) 1 and 3 network as well as canonical and non-canonical nuclear factor κ-light-chain-enhancer of activated B cell (NF-κB) signaling have been reported to be the major downstream players of CIN-induced inflammatory signaling. Although these factors are often reported to operate in separate pathways, it is becoming increasingly clear that these signaling cascades are very much intertwined (Fig. 2).

### (CIN-induced) STAT1 signaling in cancer

**Fig. 3:**
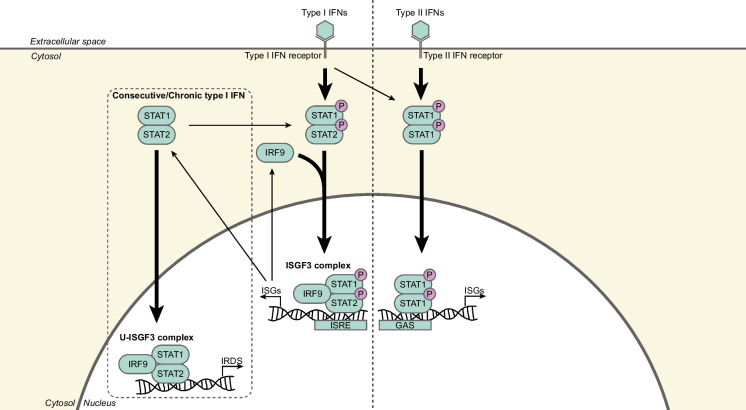
The downstream transcriptional response of STAT1 is determined by the type of stimulating IFN as well as the duration of IFN exposure. Type I IFNs (IFN ɑ and β) bind to the (tumor) cell surface type I IFN receptor. This results in activation of the type I IFN receptor-associated Janus activated kinases (JAK) tyrosine kinase 2 (TYK2) and JAK1 (not shown here). These JAKs phosphorylate STAT1 and STAT2 resulting in formation of STAT1/STAT2 heterodimers, which subsequently associate with IRF9. The formed ISGF3 complex—composed of STAT1, STAT2, and IRF9—binds to the ISREs that are present in the promoters of certain ISGs in the nucleus. As STAT1, STAT2, and IRF9 are ISGs, constitutive or chronic type I IFN exposure drives the formation of an unphosphorylated ISGF3 complex. This transcriptional complex induces the expression of IRDS. Type II IFNs (IFN γ) bind to the type II IFN receptor on the (tumor) cell surface. Both type I and II IFNs stimulate the formation of phosphorylated STAT1 homodimers that bind the GAS element in the promotor of certain ISGs. *GAS, interferon-γ-activated site; IFNs, interferons; IRDS, interferon-related DNA damage resistance signature; IRF9, interferon response factor 9; ISGF3, interferon-stimulated gene factor 3; ISGs, interferon-stimulated genes; ISRE, interferon-stimulated response elements; STAT, signal transducer and activator of transcription*

STAT1 is a central mediator of both type I (ɑ and β) and type II (ɣ) interferons (IFNs), regulating an antiviral and immune defense transcriptional response and is considered to play a central role in antitumor immunity (Avalle et al. 2012). The downstream transcriptional response of STAT1 is dependent on the type of transcriptional complex that is induced (Fig. 3) (Platanias 2005), which is largely determined by the type of stimulating IFN (Platanias 2005) as well as the duration of IFN exposure (Cheon et al. 2023). For example, type I IFNs induce the interferon-stimulated gene factor 3 (ISGF3) complex, which only binds IFN-stimulated response elements (ISREs) that are present in the promoters of certain interferon-stimulated genes (ISGs), whereas type I and II IFNs induce the transcription of ISGs with the IFN-ɣ-activated site (GAS) element in the promotor (Platanias 2005). Additionally, acute type I IFN expression drives the expression of ISGs that exert cytotoxic, antiviral effects, whereas chronic or consecutive IFN expression drives the expression of a subset of ISGs, defined as the IFN-related DNA damage resistance signature (IRDS) and whose expression correlates with resistance of cancer cells to DNA-damaging cancer therapy, extensively reviewed in (Cheon et al. 2023). Thus, the downstream consequences of (tumor) cell intrinsic STAT1 signaling are highly dependent on the context.

In the context of cancer, including tumors with CIN, STAT1 has generally been shown to exert tumor suppressor properties (Avalle et al. 2012). A decrease or loss of STAT1 activity has been reported for many cancer types (Meissl et al. 2017), including tumors with CIN (Schubert et al. 2021), and high STAT1 expression levels correlate with better clinical outcomes (Chen et al. 2013; Gordziel et al. 2013). Mechanistically, tumor cell-intrinsic STAT1-mediated tumor suppression is due to its antiproliferative effects via cell cycle inhibition and induction of cell death (Bromberg 2001; Meissl et al. 2017). Additionally, STAT1 plays a role in regulating the immunogenicity of tumor cells (Avalle et al. 2012; Meissl et al. 2017). For example, STAT1-mediated upregulation of major histocompatibility complex (MHC) class I facilitates the interaction with cytotoxic T cells but impedes natural killer cell recognition (Kaplan et al. 1998; Shankaran et al. 2001; Messina et al. 2013). The latter is in line with emerging studies that also point toward tumor-promoting and immune evasive functions of STAT1 (Meissl et al. 2017). *In vitro* studies demonstrate type II IFN-induced STAT1 activation results in upregulation of PD-L1 expression on tumor cells, thereby inhibiting T cell and natural killer cell-mediated tumor cell killing (Liu et al. 2007; Bellucci et al. 2015).

More specifically in the context of CIN, drugs or genetic drivers that decrease mitotic fidelity have been found to lead to activation of STAT1 signaling, indicated by increased levels of phosphorylated STAT1 in various cancer types, including breast cancer (Bakhoum et al. 2018; Hong et al. 2022) and acute myeloid leukemia (Jin et al. 2020). Mechanistically, Hong *et al.* found that activation of STAT1 signaling promotes cell death in breast cancer cells with induced CIN, suggesting a tumor suppressive role for STAT1 signaling in cancers with CIN (Hong et al. 2022). In alignment with this tumor suppressive role of STAT1, an *in vivo* genome-wide transposon mutagenesis screen revealed that specifically tumors that display CIN inactivate inflammatory signaling through STAT1 inactivation in combination with increased c-Myc activity compared to euploid tumors (Schubert et al. 2021). Here, STAT1 signaling in cancer cells with CIN was associated with immune cell attraction and activation, which was decreased upon loss of STAT1. In all, these studies suggest tumor suppressive roles for STAT1 signaling in cancer cells with CIN. This suggests that tumors with CIN need to overcome STAT1 signaling to survive. However, the mechanisms underlying regulation of STAT1 activity in cancer cells with CIN are still poorly understood.

### (CIN-induced) STAT3 signaling in cancer

In contrast to STAT1, STAT3 is generally described to have tumor-promoting properties, as it regulates the expression of genes involved in cell proliferation, apoptosis, and metastasis (Avalle et al. 2012). Indeed, hyperactivation of STAT3 has been reported in many cancers. The transcriptional regulatory properties of STAT3 are stimulated upon the binding of interleukin 6 (IL-6), interleukin 10 (IL-10), or growth factors, such as epidermal growth factor (EGF), fibroblast growth factor (FGF), and insulin-like growth factor (IGF), to their corresponding receptor rather than IFNs (Tolomeo and Cascio 2021). Like STAT1 signaling, the downstream transcriptional response of STAT3 is dependent on the type of transcriptional complex that is induced, which is determined by the type of stimulating cytokine (Yang and Stark 2008).

It is becoming increasingly clear that CIN and DNA damage can trigger IL-6/STAT3 signaling in various cancer types, including breast cancer (Kettner et al. 2019; Hong et al. 2022; Vasiyani et al. 2022) and ovarian cancer (Zhang et al. 2021). This is evidenced by increased expression of IL-6 as well as increased phosphorylation of STAT3 following CIN or DNA damage. Interestingly, IL-6/STAT3 pro-survival signaling appears to be important for the survival of cancer cells with CIN, as inhibition of IL-6/IL-6R signaling by the IL-6R inhibitor tocilizumab decreased proliferation and/or increased cell death of ovarian, breast, and lung cancer cell lines *in vitro* as well as *in vivo*. The mechanism underlying CIN-induced IL-6/STAT3 signaling is dependent on cGAS/STING and non-canonical NF-κB signaling (Hong et al. 2022). However, how these pathways precisely interact in the context of CIN is still poorly understood. Intriguingly, in the CIN^+^ prostate cancer cell line DU-145, IL-6/STAT3 signaling was found to inhibit STING activity, as these cells were only responsive to the STING agonist 2′3′-cGAMP when IL-6 or JAK/STAT3 signaling was inhibited, suggesting that STING is regulated by IL-6/STAT3 signaling upstream (Suter et al. 2021). Together, these studies demonstrate the complicated and intertwined relationship between (cGAS)/STING activity and STAT3 signaling in cancers with CIN.

### (CIN-induced) NF-κB signaling in cancer

Chronic activation of NF-κB affects multiple cellular processes in cancer including inflammation, transformation, proliferation, angiogenesis, invasion, metastasis, chemoresistance, and radiotherapy resistance and can lead to “NF-κB addiction” of cancer cells (Chaturvedi et al. 2011). Activation of NF-κB is mediated through diverse stimuli that originate from the tumor (immune) microenvironment, such as pro-inflammatory cytokines IL-1, TNF, and IL-23, which differ between canonical NF-κB and non-canonical NF-κB (Li et al. 2010; Liu et al. 2017). Furthermore, STING can also activate canonical NF-κB signaling through an interaction with the IκB kinase (IKK) complex (Fig. 1) (Hoesel and Schmid 2013).

CIN has been shown to activate canonical NF-κB signaling, involving p50 and p65, as well as the non-canonical NF-κB signaling, involving p52 and RelB (Figs. 1 and 2), and was found to promote tumorigenesis in multiple studies (Hong et al. 2019; Beernaert and Parkes 2023). NF-κB signaling induced by CIN has been associated with an oncogenic role in multiple studies. For instance, Bakhoum *et al.* found that CIN promotes metastasis driven by STING-induced non-canonical NF-κB signaling (Bakhoum et al. 2018). In line with this, a positive correlation between CIN, NF-κB (target) mRNA expression levels, and lymph node metastasis was observed in oral squamous cell cancer from TCGA data (Biswas et al. 2019). Furthermore, non-canonical NF-κB signaling was found to be required for the survival of cancer cells with induced CIN phenotypes (Hong et al. 2022). On the other hand, tumor suppressive effects have been reported as well. For instance, acute induction of CIN was found to suppress invasive behavior of several (cancer) cell lines, which coincided with activation of non-canonical NF-κB and downstream inflammatory signaling (Vasudevan et al. 2020). Furthermore, non-canonical NF-κB signaling was found to promote genome integrity in diffuse large B cell lymphoma by preventing CIN and DNA damage (Ramachandiran et al. 2015). In senescent cells with complex aneuploid karyotypes, NF-κB signaling contributes to natural killer cell-mediated elimination. However, natural killer cell-mediated elimination was not induced in aneuploid cancer cell lines, despite upregulation of NF-κB signaling, suggesting that aneuploid cancer cells circumvent immune activation (Santaguida et al. 2017; Schubert et al. 2021; Wang et al. 2021). While these studies reveal clear interactions between CIN phenotypes and NF-κB signaling, the type of interaction is likely context-specific. Therefore, to better understand under which conditions NF-κB signaling is tumor suppressive or oncogenic in cancers with CIN requires further work.

## The consequences of CIN-induced cell-intrinsic inflammatory signaling for antitumor immunity

Although acute tumor cell-intrinsic inflammatory signaling has traditionally been considered to promote immune cell surveillance (Hong et al. 2019; Ablasser and Hur 2019), emerging evidence is pointing toward a more complex relationship between cell-intrinsic inflammatory signaling and antitumor immunity in tumors with CIN. This presents challenges for clinical targeting of CIN-induced cell-intrinsic inflammatory signaling to enhance antitumor immunity.

### Evasion of CIN-induced antitumor immunity

Cell-intrinsic inflammatory signaling was initially reported to increase the immunogenicity of tumors with CIN via cGAS/STING-mediated activation of the type I IFN response followed by STAT1 signaling, thereby driving immune infiltration (Tripathi et al. 2019; Schubert et al. 2021). Therefore, efforts were made to activate this signaling using, for instance, STING agonists in clinical trials, though not (yet) specifically for CIN^+^ cancers (Le Naour et al. 2020). However, the relationship between CIN and immune surveillance might be more complicated than originally anticipated. Davoli *et al.* found that human cancers with extensive aneuploidy are associated with markers of immune evasion rather than immune surveillance. These markers involved low expression of genes associated with adaptive immunity, cytotoxic activities mediated by cytotoxic T cells and NK cells, and decreased activity of pathways related to an active immune response and a cytokine-rich microenvironment (Davoli et al. 2017). Similarly, *in vivo* propagation of CIN^+^ tumors in immuno-proficient mice, but not immuno-deficient mice, led to decreased inflammatory signaling in the CIN^+^ cancer cells evidenced by a reduced IFN response and decreased MHC class I antigen presentation (Tripathi et al. 2019). These findings agree with another study in which the cancer drivers were compared between CIN^−^ and CIN^+^ tumors in mice, which found that CIN^+^ but not CIN^−^ cancers alleviate STAT1 and IFN inflammatory signaling (Schubert et al. 2021). Indeed, STAT1 is known to upregulate expression of MHC class I (Kaplan et al. 1998; Messina et al. 2013; Shankaran et al. 2001), and therefore, alleviation of STAT1 signaling might well explain decreased MHC class I antigen presentation in CIN^+^ cancers. Altogether, these findings suggest that while CIN initially might promote tumor inflammation, ultimately CIN cancers find a way to circumvent this inflammatory response to prevent immune clearance. Undermining these immune-evasive mechanisms might thus provide a powerful strategy to treat CIN^+^ cancers.

### Immunotherapies to enhance antitumor immunity in cancers with CIN

The finding that CIN triggers a cell-intrinsic inflammatory response that modulates the immune microenvironment urged the field to investigate the effect of immunomodulatory therapies as means to target cancers with CIN. Since cGAS/STING signaling plays an important role in enhancing the antitumor effects of immune checkpoint inhibitors (ICI) (Jiang et al. 2020), ICIs might act synergistically in combination with CIN to elicit an antitumor immune response. However, so far the results of these studies have been inconclusive. For example, patients with high-grade serous ovarian carcinoma tumors that displayed high CIN (and DNA damage repair gene deficiency) did not benefit from ICIs targeting the PD1/PD-L1 axis, despite these tumors being highly immunogenic (Shakfa et al. 2022). These findings agree with another study, which investigated the association between the 70-gene CIN signature and the response to ICIs in a melanoma and urothelial cancer cohort but did not find a predictive value for the 70-gene CIN signature regarding treatment outcome (Wu et al. 2020). In contrast, Davoli *et al.* identified somatic copy number alterations (CNA), *i.e.*, segmental aneuploidies, as a predictor for the survival of patients after immunotherapy in a melanoma cohort; the somatic CNA levels were lower in patients with long-term survival compared to patients with short-term survival (Davoli et al. 2017). A similar trend for tumor CNA levels as a predictor of prognosis following ICI treatment was identified for cancers characterized by lower tumor mutational burden (Spurr et al. 2022). However, careful consideration on the cutoff during CNA calling and larger data samples are necessary to obtain a higher predictive value for patient survival following ICI treatment (Chang et al. 2023). Therefore, before CIN and/or CNAs can become an important predictor for patient survival and/or stratification for immunotherapy responsiveness, further evaluation of the clinical utility and underlying molecular mechanisms of these factors is necessary.

## Conclusion and outlook

In this review, we discussed the complex relationship between CIN and inflammation in cancer, the intratumoral mechanisms involved in CIN-induced inflammation, and their impact on the survival of tumors with CIN. It is becoming increasingly clear that cell-intrinsic inflammatory signaling in cancer cells with CIN can have tumor-promoting as well as tumor-suppressive effects mediated by STAT3 (and NF-κB) and STAT1 signaling, respectively. These antagonistic effects of cell-intrinsic inflammatory signaling might explain why cancers rarely show loss of function mutations of cGAS and STING (Bakhoum and Cantley 2018), but rather epigenetic silencing (Konno et al. 2018). The balancing act between pro-survival STAT3 signaling and pro-death STAT1 signaling downstream of cGAS/STING signaling resulting from CIN allows cancer cells to cope when the insult is not too severe, while still allowing cells to induce apoptosis and promote immune clearance when needed. As CIN fuels karyotypic heterogeneity during tumor cell evolution, CIN will likely promote selection of karyotypes that promote activity of pro-tumorigenic non-canonical NF-κB and STAT3 signaling and inhibit tumor-suppressive STAT1 signaling programs. As such, CIN can drive the switch from immune surveillance to immune evasion, of which the former was induced by CIN to begin with.

Importantly, the interaction between CIN^+^ cancer cells and their immune microenvironment might well provide new opportunities to target cancers with CIN. To develop such therapies, we first need to better understand the many downstream effects of this inflammatory response, their exact time scale, and how they interact. Therefore, it is key to further unravel how and when exactly cancer cells with CIN trigger an inflammatory response and how cancers with CIN abrogate this immune response. With what is known now, one would predict that promoting STAT1-mediated cell death following CIN while blocking STAT3-mediated cell survival could be a powerful strategy to kill cells with (induced) CIN. Alternatively, reactivation of STAT1-mediated immune signaling in CIN^+^ cancers could provide a strategy to selectively treat aneuploid cancers. However, before such therapies will reach the clinic, further *in vivo* work and clinical trials confirming these hypotheses are required.

## Data Availability

Not applicable.
